# Nitrous oxide abuse unmasking anti-phospholipid syndrome in a 24-year-old male with cerebral venous thrombosis and pulmonary thromboembolism: a case report

**DOI:** 10.1186/s12883-025-04237-x

**Published:** 2025-05-26

**Authors:** Sojung Yoon, Dong Yu Kim, Min Kyung Chu

**Affiliations:** Department of Neurology, Severance Hospital, Yonsei University College of Medicine, Seoul, South Korea

**Keywords:** Nitrous oxide, Vitamin B_12_ deficiency, Cerebral venous thrombosis, Anti-phospholipid syndrome, Case report

## Abstract

**Background:**

The abuse of nitrous oxide (N_2_O) as a recreational drug has become a growing concern, manifesting in various medical complications. Here, we report the first Korean case of cerebral venous thrombosis (CVT) and pulmonary thromboembolism (PTE) caused by nitrous oxide abuse and anti-phospholipid syndrome.

**Case presentation:**

A 24-year-old Korean man studying abroad in the USA presented to the emergency department with altered sensorium, gait disturbance, and involuntary leg movements. His history revealed escalating nitrous oxide inhalation from occasional use to daily intake of 100 balloons over six months. The patient’s symptoms were initially attributed to cobalamin deficiency due to N_2_O abuse, leading to hyperhomocysteinemia and subsequent venous thrombosis, particularly CVT and PTE. However, the presence of Lupus Anticoagulant (LA) indicated a potential autoimmune or inflammatory process contributing to thrombotic complications, complicating the diagnosis. The patient’s treatment involved cobalamin supplementation, anticoagulation therapy, and addressing associated substance abuse and depression. Notably, subsequent tests revealed persistent positive LA, highlighting the complexity of the case and the multifactorial nature of N_2_O-induced complications.

**Conclusions:**

The mechanisms underlying N_2_O-induced thrombotic complications are elucidated, primarily involving the oxidation of cobalamin to its inactive form, leading to hyperhomocysteinemia and endothelial dysfunction. The association with LA suggests a potential autoimmune or inflammatory component, adding another layer of complexity to pathophysiology. This case underscores the importance of recognizing and managing the systemic thrombotic risk associated with N_2_O abuse. It highlights the need for heightened vigilance in diagnosing and managing associated complications, including CVT, and emphasizes the importance of comprehensive diagnostic and treatment approaches addressing both medical and psychiatric aspects. In conclusion, this case serves as a poignant reminder of the serious medical consequences of N_2_O abuse and underscores the importance of early recognition, comprehensive management, and ongoing surveillance to mitigate its adverse effects on individual health and public well-being.

## Background

Nitrous oxide (N_2_O) is inhalational anesthetic agent widely used in dentistry and minor medical procedures [1]. Nitrous oxide exerts rapid-onset anxiolytic and hallucinogenic effects, typically beginning within 10 s of inhalation and dissipating within minutes [2]. The recreational use of nitrous oxide has surged in recent years. N_2_O can increase the risk of vitamin B_12_ deficiency as it irreversibly binds and inactivates vitamin B_12_ [3, 4]. Its chronic or heavy abuse can lead to complications such as peripheral neuropathy, subacute combined degeneration, bone marrow suppression, and even cerebral venous thrombosis (CVT) [5]. Hyperhomocysteinemia, a consequence of vitamin B12 deficiency, is a recognized risk factor for thrombosis, which overlaps mechanistically with the hypercoagulable state observed in anti-phospholipid syndrome (APS). Although APS is an autoimmune disorder characterized by thrombotic events and persistent antiphospholipid antibodies, environmental or drug-related triggers may unmask latent disease in predisposed individuals [6]. Pulmonary embolism with nitrous oxide inhalant abuse case with a single positive Lupus Anticoagulant (LA) test has been reported once [7], but it was not confirmed and the patient was COVID-19 positive. To the best of our knowledge, this is the first reported case of nitrous oxide abuse unmasking APS in a patient diagnosed with CVT and pulmonary thromboembolism (PTE).

## Case presentation

**Fig. 1:**
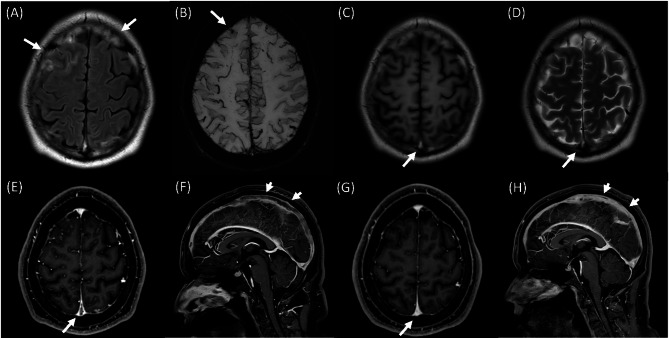
Brain MR images of the patient on admission to the hospital, and 2 months after admission. **(A)** Brain MR FLAIR image revealing sulcal hyperintensity (arrows), suggesting cortical SAH. **(B)** SWI image displaying dark signal intensity, highlighting cortical subarachnoid hemorrhage at the bilateral frontal sulci (arrow) **(C)** T1-weighted and **(D)** T2-weighted images depicting hyperintensity at the superior sagittal sinus and adjacent cortical veins (arrows), suggesting CVT **(E-F)** Contrast enhanced T1-weighted images revealing filling defects within the superior sagittal sinus, where blood flow is blocked by the blood clot (arrows). **(G-H)** Follow-up MR performed two months after admission and ongoing anticoagulation therapy demonstrating a significant reduction in the extent of venous sinus thrombosis (arrows) FLAIR: fluid attenuated inversion recovery; MR: magnetic resonance; SWI: susceptibility weighted imaging; CVT: cerebral venous thrombosis

**Fig. 2:**
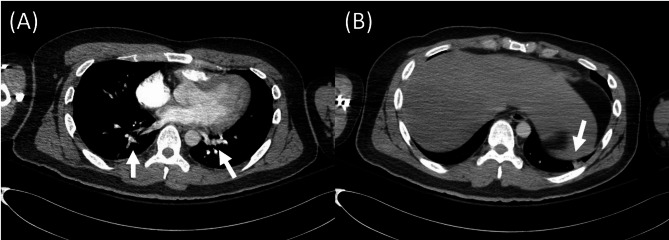
Pulmonary CT image of the patient on admission to the hospital. **(A)** Pulmonary CT scan revealing pulmonary thromboembolism involving the segmental and subsegmental pulmonary arteries in both lungs (arrows). **(B)** Patchy subpleural ground-glass opacity and consolidation in left basal lung, findings suggestive of either pulmonary infarction or pneumonia (arrow) CT: computed tomography

We present a case of a 24-year-old Korean man studying abroad in the USA, who presented to the emergency department with altered sensorium. He had no significant medical history but a history of recreational nitrous oxide inhalation. His use of nitrous oxide escalated from occasional use to a daily intake of 100 balloons over six months. He was admitted to a hospital in the USA for one day due to intermittently confused mentality, gait disturbance and involuntary leg movement. His urine drug level, routine CBC, chemistry, chest X-ray, brain CT scan and electroencephalography all showed normal results, according to his parents’ account. Since he was discharged against medical advice, his medical information could not be retrieved, and his medical records in the USA were entirely based on his parents’ statements. He showed initial improvement with cobalamin supplementation for B_12_ deficiency but returned to Korea immediately after discharge. In Korea, his symptoms relapsed several days later, despite no longer abusing N_2_O. He denied using any drugs other than nitrous oxide during the initial evaluation. He had no headache, dizziness, nausea, vomiting, diplopia, dyspnea and chest pain. He exhibited intermittent confusion, altered sensorium, and involuntary leg movements, which appeared more consistent with agitation rather than jerky or myoclonic movements. The neurological examination indicated normal cranial nerve function, as well as intact proximal and distal muscle strength. Sensory perception, including touch, pain, temperature, vibration, and proprioception, was also normal. There was no ataxia observed during the finger-to-nose and heel-to-shin test, and the deep tendon reflexes were normal. His gait appeared grossly normal, despite some difficulty with balance. He scored 22 out of 30 on the Korean version of the mini-mental state examination-2 (K-MMSE-2, license was bought from Inpsyt, Inc., a branch of Hakjisa publisher), displaying impairments in memory, orientation, and attention. Routine laboratory tests were repeated in Korea, along with a chest X-ray and electrocardiogram, all of which showed normal findings. His urine drug levels for acetaminophen, amphetamines, benzodiazepines, zolpidem, and ethanol were negative. Nitrous oxide levels were not measurable in Korea, so results were unknown. However, mildly elevated levels of CRP (16.9 mg/dL; normal < 8 mg/dL) and ESR (70 mm/hr; normal < 15 mm/hr) were noted without a specific focus of inflammation. His coronavirus disease-19 PCR tests were negative. He was positive for LA, while tests for Rheumatoid factor, antinuclear antibody, antineutrophil cytoplasmic antibody, anti-β2-glycoprotein I IgG and IgM and anti-cardiolipin antibody, were negative. His laboratory results demonstrated elevated levels of C3 (206.8 mg/dL; normal 90-180 mg/dL) and C4 (59.83 mg/dL; normal 10-40 mg/dL), which may reflect an underlying inflammatory activity. In addition, protein C (138%; normal 70–130%), protein S (> 150%; normal 62–150%), and antithrombin III (129%; normal 80–120%) were all elevated, supporting a hypercoagulable state while providing evidence against protein C or S deficiency. Nevertheless, d-dimer (3,577 ng/mL; normal < 243ng/mL) and homocysteine (30.12 mmol/L; normal < 15.0 mmol/L) levels were elevated, while vitamin B_12_ (695 pg/mL), folate (20.11 ng/mL) and methylmalonic acid levels (2.41 umol/L) were within normal ranges. A spinal tap was performed to assess intracranial pressure and central nervous system inflammation. The results indicated elevated intracranial pressure (260 mm CSF), with normal cell count, protein, glucose, lactate, and adenosine deaminase levels. Further evaluation with electroencephalography was conducted to assess altered sensorium and possible epilepsy, given his involuntary leg movements. The findings demonstrated continuous diffuse theta to delta dominant background slowing. Brain magnetic resonance (MR) imaging provided critical insights, identifying venous sinus thrombosis at the superior sagittal sinus and adjacent cortical veins, along with cortical subarachnoid hemorrhage (SAH) at bilateral frontal sulci (Fig. 1A-F). As the diagnostic workup continued, a subsequent chest CT scans unveiled PTE, further indicating a systemic thrombotic process (Fig. 2A, B). However, he did not have any symptoms of PTE, except occasional sinus tachycardia up to 130-150 bpm. Echocardiography was performed to assess for right ventricular strain, but no abnormalities were observed. The patient reported no family history of autoimmune or rheumatic diseases. We recommended genetic testing for thrombotic conditions, including methylenetetrahydrofolate reductase (MTHFR) polymorphism; however, the patient declined testing.

Anticoagulation therapy was initiated with targeting an aPTT range of 50 to 60 to mitigate the risk of hemorrhagic complications, given the presence of SAH. Heparinization was maintained for five days, with the aPTT reaching the target range for three days, beginning on the second day after initiation. Heparinization was discontinued due to clinical improvement, including regained ability to communicate and improved mental status. The anticoagulation regimen was then transitioned to apixaban at a dose of 5 mg twice daily before discharge. In total, the patient received anticoagulation therapy for ten days. After 2 months of anticoagulation and replacement therapies using mecobalamin at 0.5 mg three times per day and folic acid at 0.5 mg per day, his abnormal movements, gait, and cognition (MMSE, 30/30) have normalized. About two months after admission, brain MR venography revealed a reduction in the extent of cortical SAH and venous sinus thrombosis (Fig. 1G-H). Subsequent LA tests conducted at 12-week intervals remained positive, confirming anti-phospholipid syndrome (APS). According to the 2023 ACR/EULAR antiphospholipid syndrome (APS) classification criteria [8], the patient meets the clinical criteria based on venous thromboembolism and the laboratory criteria with persistent LAC positivity, supporting a diagnosis of APS. Additionally, the 1999 revised Sapporo criteria [9] and the 2006 revised Sydney criteria [10] recommend confirming LAC positivity at intervals greater than 12 weeks, a requirement fulfilled by the patient’s laboratory findings. He reported no specific adverse events. As evidence suggests superior treatment outcomes with warfarin compared to non-vitamin K antagonist oral anticoagulants in patients with APS, we initially planned to transition the patient from apixaban to warfarin following the positive LA result [11]. However, the patient declined warfarin therapy due to his need to return to the United States. Consequently, treatment with a non-vitamin K antagonist oral anticoagulant was continued. Additionally, the patient reported difficulty adhering to a twice-daily medication regimen. As a result, apixaban was switched to once-daily rivaroxaban therapy four months after discharge, with plans to reduce the dose given the absence of thrombotic symptom relapse. However, the patient was lost to follow-up after March 2025.

## Discussion and conclusions

The case highlights a potential association between recreational nitrous oxide inhalation and the development of neurological complications, including venous sinus thrombosis and cortical SAH. The diagnosis of APS was confirmed, which is one of the risk factors for CVT. To our knowledge, this is the first documented case in which nitrous oxide abuse revealed underlying APS in a patient who subsequently developed both CVT and PTE.

This case report has some limitations. First, the initial levels of vitamin B_12_, homocysteine, and methylmalonic acid were not obtained. Given the patient’s history of nitrous oxide abuse and the treatment he received, it is likely that his initial vitamin B_12_ level was low, accompanied by elevated homocysteine and methylmalonic acid levels. However, even if his initial vitamin B_12_ levels were within the normal range, functional vitamin B_12_ deficiency cannot be excluded, as cases of nitrous oxide abuse with normal serum vitamin B_12_ levels have been previously reported [12]. Second, the patient declined genetic analysis for factors associated with increased coagulability, such as MTHFR polymorphism [13] or prothrombin gene mutation [14]. Third, it is unclear whether CVT is attributable to APS or N_2_O abuse. The clinical criteria for APS are not applicable if a more likely alternative explanation is identified [8]; however, APS is a well-recognized risk factor for thrombosis, including CVT [15]. Although N₂O-associated thrombosis has predominantly been reported in case reports and case series, we concluded that the patient had underlying APS, with N₂O abuse serving as an aggravating factor that contributed to the development of CVT and pulmonary embolism. There has been a reported case of APS diagnosed in a patient with cocaine abuse, presenting with retiform purpura [16].

Several cases of N_2_O abuse with CVT have been reported to date. There have been reports of young adults with a history of nitrous oxide abuse developing CVT despite the absence of other known thrombophilia risk factors in France [17], Taiwan [18], Thailand [19], and two cases in China [20, 21]. Additionally, a patient in the Netherlands with a history of N_2_O abuse was found to have a heterozygous prothrombin G20210A (factor II) gene mutation [5], while one patient each in China and the United States with a similar history tested positive for significant MTHFR polymorphisms [13, 22]. Another case report from the United States described a young male diagnosed with PTE who had a history of nitrous oxide abuse and tested positive for COVID-19 via PCR [7]. The patient also had a positive LA test; however, follow-up testing at 12 weeks was not reported. Given the well-established association between COVID-19 and various thrombotic complications, chronic anticoagulation was not initiated in this patient [23].

It has been proposed that nitrous oxide oxidizes cobalamin to its inactive form, thus depleting the active methylcobalamin, which is an essential cofactor for methionine synthesis [24]. This depletion of methionine and subsequent accumulation of homocysteine could contribute to the development of homocysteinemia, which has been associated with venous thrombosis in previous studies [25]. Such homocysteinemia could potentially lead to CVT as well as PTE. In a 2003 case-control study involving 121 patients with a first episode of CVT, hyperhomocysteinemia was diagnosed in 33 patients (27%). In contrast, it was identified in only 20 control subjects (8%) [26]. Additionally, a study has shown that 32% of patients with CVT had hyperhomocysteinemia without any other identified thrombotic risk factors, such as protein S deficiency, protein C deficiency, MTHFR mutation, antinuclear antibodies, or Factor V Leiden mutation [27]. Several meta-analyses have also demonstrated an association between hyperhomocysteinemia and venous thrombosis [25, 28, 29].

There is a case report with a 25-year-old woman who had a history of nitrous oxide abuse and presented with CVT and SAH [30]. While there isn’t a well-established and direct mechanism linking nitrous oxide abuse to SAH, it is plausible that an increased risk of vascular damage and clot formation could potentially contribute to the rupture of blood vessels and lead to SAH. Additionally, hyperhomocysteinemia could result in elevated levels of reactive oxygen species, potentially causing endothelial dysfunction [31, 32]. Inhalation of nitrous oxide is a well-documented cause of vitamin B_12_ deficiency [4], which disrupts the vitamin B_12_-dependent pathways involved in methionine and folate metabolism [33]. Numerous in vitro and in vivo studies have demonstrated the direct cytotoxic effects of elevated homocysteine levels on endothelial cells, further underscoring the clinical significance of this metabolic disruption [34–36]. Hyperhomocysteinemia leads to the accumulation of the endogenous nitric oxide synthase inhibitor asymmetric dimethylarginine [37] and inhibits the expression of the antioxidant enzyme cellular glutathione peroxidase. This results in increased reactive oxygen species, which inactivate nitric oxide and contribute to endothelial function [38].

Psychiatric symptoms are known to occur in relation to nitrous oxide use, although the exact pathophysiology of nitrous oxide-induced psychosis remains unclear [39]. In our case, the patient exhibited involuntary leg movements, possibly attributed to nitrous oxide psychosis or increased intracranial pressure. Our patient, who was a heavy nitrous oxide user, experienced a combination of intracranial hypertension, CVT, and pulmonary infarct.

This represents the first reported case in Korea of an APS patient with N_2_O abuse associated with CVT, emphasizing the importance of recognition and intervention. This case underscores the potential multifactorial pathogenesis of CVT and pulmonary embolism in the setting of APS and nitrous oxide abuse. While APS was confirmed based on established diagnostic criteria, N₂O abuse likely acted as a significant aggravating factor, contributing to disseminated thrombotic complications through mechanisms such as hyperhomocysteinemia and vitamin B_12_ deficiency. The case emphasizes the need for heightened clinical awareness regarding the thrombotic and neuropsychiatric risks associated with recreational N₂O use, particularly in patients with underlying prothrombotic conditions. Treatment addressing depression and substance abuse may be warranted in such cases.

## Data Availability

No datasets were generated or analysed during the current study.

## Abbreviations

N_2_O: Nitrous oxide
CVT: Cerebral venous thrombosis
LA: Lupus Anticoagulant
PTE: Pulmonary thromboembolism
CBC: Complete blood count
CT: Computed tomography
MMSE: Mini-mental state examination
CRP: C-reactive protein
ESR: Erythrocyte sedimentation rate
CSF: Cerebrospinal fluid
MR: Magnetic resonance
SAH: Subarachnoid hemorrhage
aPTT: Activated partial thromboplastin time
APS: Anti-phospholipid syndrome
